# Towards a Better Prognosis in Patients with Systemic Sclerosis-Related Pulmonary Arterial Hypertension: Recent Developments and Perspectives

**DOI:** 10.3390/jcm13195834

**Published:** 2024-09-30

**Authors:** Maria Boutel, Athanasia Dara, Alexandra Arvanitaki, Cleopatra Deuteraiou, Maria Mytilinaiou, Theodoros Dimitroulas

**Affiliations:** 1 Fourth Department of Internal Medicine, Hippokration University Hospital, School of Medicine, Aristotle University of Thessaloniki, 54642 Thessaloniki, Greece; mmpoutelag@gmail.com (M.B.); dara.athanasia@yahoo.gr (A.D.); deutereou@hotmail.com (C.D.); mariamytilinaiou@gmail.com (M.M.); dimitroul@hotmail.com (T.D.); 2 Adult Congenital Heart Centre and National Centre for Pulmonary Hypertension, Royal Brompton and Harefield Hospitals, Guy’s and St Thomas’s NHS Foundation Trust, Imperial College, London SW3 6NP, UK; 3 First Department of Cardiology, AHEPA University Hospital, School of Medicine, Faculty of Health Sciences, Aristotle University of Thessaloniki, 54636 Thessaloniki, Greece

**Keywords:** precapillary pulmonary hypertension, systemic sclerosis, prognosis, screening, pharmacotherapy, survival

## Abstract

Precapillary pulmonary hypertension (PH) is a significant complication of systemic sclerosis (SSc). It represents one of the leading causes of morbidity and mortality, correlating with a significantly dismal prognosis and quality of life. Despite advancements in the management of patients with pulmonary arterial hypertension associated with SSc (SSc-PAH), no significant improvement has been reported in survival of patients with precapillary SSc-PH associated with extensive lung parenchyma disease. International expert consensus and guidelines for the management of PH recommend annual screening of SSc patients for early detection of pre-capillary PH. The implementation of screening algorithms capable of identifying patients with a high likelihood of developing PH could help limit unnecessary right-heart catheterization procedures and prevent significant delay in diagnosis. Furthermore, early initiation of up-front combination targeted therapy in patients with PAH has shown increase in survival rates, indicating that timely and aggressive medical therapy is key for stabilizing and even improving functional class, hemodynamic parameters and 6 min walking distance (6MWD) in this population. Further research is warranted into the benefit of PAH-targeted therapies in patients with PH associated with lung disease. Lastly, we discuss the potential role of immunosuppression using biologic agents in the therapeutic management of precapillary PH in SSc patients.

## 1. Introduction

Systemic sclerosis (SSc) is a chronic multisystem autoimmune connective tissue disease characterized by vasculopathy, inflammation and fibrosis of the skin and internal organs, as well as the presence of serum autoantibodies [1]. SSc has the highest burden of death among rheumatic diseases [2] and is expressed through a localized or a systemic phenotype, which can occur in two forms: limited and diffuse [3]. Precapillary pulmonary hypertension (PH) is hemodynamically defined as mean pulmonary artery pressure (mPAP) > 20 mmHg, pulmonary wedge pressure ≤ 15 mmHg and pulmonary vascular resistance > 2 WU. Pulmonary arterial hypertension (PAH) (group 1) or PH associated with lung parenchymal disease and/or hypoxia (group 3) are the most frequently encountered precapillary PH phenotypes in patients with SSc [4]. Precapillary PH is the second SSc-related respiratory manifestation [5] and one of the primary causes of hospitalization and mortality among SSc patients [6].

SSc-PAH patients, who represent 10% of the SSc population, are a significant current challenge for clinicians, as they exhibit a 3-year survival rate of 20–70%, varying according to WHO functional class categorization [7,8]. The frequent co-existence of interstitial lung disease (ILD) and PH (PH-ILD) in SSc, which may be identified in 2–18% of SSc patients, conveys higher mortality rates and further aggravates survival [9]. SSc-ILD can manifest in a variety of ways, from mild non-progressive lung involvement to severe respiratory failure, and it is accountable for 35% of SSc-related deaths [10]. According to research findings, the 5-year survival rate of patients with SSc-related PH-ILD is lower, at approximately 30% to 40%, compared to SSc-PAH patients [9,11]. PAH and group 3 PH carry a grim prognosis, poorer than that of idiopathic PH and other connective tissue disease associated-PH (CTD-PH). However, in the last decade, numerous studies and randomized controlled trials have been carried out aiming at improving morbidity and mortality rates in this population [12]. Screening methods and effective risk stratification techniques such as the DETECT algorithm [13], novel therapeutic choices, and advanced therapeutic strategies like monotherapy or combination therapy with endothelin receptor antagonists (ERAs), phosphodiesterase 5 (PDE5) inhibitors and prostacyclin analogues, according to the individual’s risk level and the presence of cardiopulmonary comorbidities [4], have been established throughout recent years. 

However, it is still unclear if these improvements in diagnosis and treatment of PAH and PH-ILD have increased survival. Existing studies have shown contradictory results [14,15,16], with recent real-world data indicating a poor survival in patients with SSc-PH [17]. The aim of the current review is to summarize the recent screening strategies targeting early diagnosis of precapillary PH in SSc and the current advances in pharmacotherapy and their impact on the survival prospects of these patients.

## 2. Pathophysiology

Τhe cornerstone of SSc pathogenesis is vascular injury. The vascular hallmarks of scleroderma include telangiectasia, Raynaud’s phenomenon, capillary abnormalities as revealed by nailfold capillaroscopy, digital ulceration, gangrene with digit amputation and extensive vascular pathology, observed in all affected organs, such as scleroderma renal crisis and PH. Endothelial damage, capillary impairment and an imbalance of inflammatory mediators that affect the pulmonary vasculature are the main components of the SSc-PAH pathophysiology (group 1), where small to medium-sized pulmonary arterioles are predominantly affected [18]. Vascular endothelial cells may malfunction due to environmental or genetic factors, which would result in their overactivation [19]. Vasomotor compounds (nitric oxide, prostacyclins, and prostaglandins), secreted by the endothelium, influence coagulation and fibrinolysis, regulate inflammatory processes, and alter the permeability of the vessel wall. The inflammatory cells that are drawn in, as a result of these processes [20], lead to an excessive proliferation of smooth muscle cells and vascular inner membrane, activation of fibroblasts and the disorganized production of the extracellular matrix (ECM) [21,22,23]. Vascular pulmonary remodeling is facilitated by elevated production of proliferative mediators and vasoconstrictors, while reduced synthesis of vasoactive mediators alters vascular tone. Four primary pathophysiologic pathways are involved in the development of PAH: Endothelin-1 (ET-1), nitric oxide, prostacyclins and signaling mediated by the anti-proliferative bone morphogenetic protein receptor type II (BMPR-II) [24,25,26,27]. In patients with SSc-PAH, ET-1 is overexpressed in both plasma and lung tissue, and its expression is negatively correlated with survival in PAH [28].

Inflammation and autoimmunity have a predominant role in the development and course of PAH, in connective tissue disorders. From a pathological viewpoint, pulmonary arteries of patients suffering from SSc-PAH are infiltrated by inflammatory cells, such as macrophages and lymphocytes. In addition, the serum of SSc-PAH patients has also been reported to include higher levels of proinflammatory cytokines and antinuclear antibodies (ANA) [29]. According to data from animal models, B cells may promote pulmonary vascular damage and remodeling by producing antibodies against endothelial cells. Additionally, cytotoxic T lymphocytes may be involved in the process of pulmonary arterial muscularization, vascular intimal fibrosis, medial hypertrophy, and adventitial thickening [30]. Immunological activation and inflammatory processes appear to be pivotal players in the development of SSc-PAH, leading to increased levels of pulmonary vascular resistance, pulmonary arterial pressure, and right ventricular pressure overload [31].

SSc-related PH-ILD is characterized by angiogenesis/neovascularization, fibroblast proliferation and overabundance of extracellular matrix formation. The resultant state of respiratory tissue hypoxia and the vascular alterations lead to vasoconstriction [32]. Sustained or repeated exposure to hypoxia results in increased muscularization of the pulmonary vascular bed and remodeling of the distal pulmonary circulation, which is only partially reversed on correction of hypoxia [33]. SSc-related PH-ILD also exhibits increased levels of mediators known to play a key role in the genesis of idiopathic PAH, such as platelet-derived growth factor, transforming growth factor beta, ET-1, and tumor necrosis factor alpha. Consequently, these mediators may have a “crossover” effect on the pulmonary vasculature [34]. Cardiac impairment may occur as a result of these mechanisms, leading to increased pulmonary arterial pressure and pulmonary vascular resistance, resulting in greater cardiac and pulmonary impairment and, therefore, to higher mortality rates than isolated SSc-PAH [35,36]. Due to their profile and unfavorable outcomes, SSc-related PH-ILD patients require early detection and aggressive therapy combined for ILD and PH to stabilize pulmonary function [37]. 

The co-existence of comorbidities in precapillary SSc-PH patients impacts the prognosis and mortality rates. Diastolic dysfunction, which is a predecessor of heart failure with preserved ejection fraction [38], appears in 18% to 62% of SSc patients, and significantly increases mortality rates [39,40]. Atherosclerosis and diabetes mellitus may also affect SSc patients and increase mortality risk to levels similar to those of other connective tissue disorders, notoriously known for their increased cardiovascular risk, such as rheumatoid arthritis [41,42]. Myocardial involvement, diastolic dysfunction, atherosclerosis and left heart disease represent a great proportion of the precapillary SSc-PH population, with very poor outcomes and higher fatality risk when compared to SSc-PAH patients [43,44]. Physicians should be also alert for patients with a more limited cutaneous phenotype (Raynaud’s syndrome, sclerodactyly, telangiectasias, vasculopathy, ACA positivity, etc.), as these patients are at high risk of developing pulmonary hypertension and could manifest subclinical PH, while being non-dyspneic [36].

## 3. Precapillary PH Screening and Diagnosis in SSc

### 3.1. Screening and Diagnosis of Group 1 PH in SSc

The prognosis for SSc-PAH is generally dismal, as it remains alarmingly high compared to that of idiopathic PAH [16,45,46]. Screening programs for the early detection of SSc-PAH have been established and endorsed by international PH guidelines, to aid early diagnosis and management of PAH in SSc patients [4].

Early detection of symptoms and diagnosis of PAH in patients with SSc enables the prompt initiation of treatment at a more premature stage when therapies are more effective [47,48]. In order to achieve earlier diagnosis, annual screening is recommended for asymptomatic SSc patients [13,49]. A prospective study of 599 patients conducted in 21 SSc centers in France highlights the clinical value of early PAH detection with regards to prevention of hemodynamic impairment and survival improvement [50]. Patients without severe pulmonary function impairment underwent Doppler echocardiography, and those with a peak velocity of tricuspid regurgitation > 3 m/s or 2.5–3 m/s and concomitant unexplained dyspnea proceeded to right heart catheterization (RHC). Of the above-mentioned patients, 29 had known PAH and 33 had suspected PAH, based on Doppler echocardiography, and were referred for RHC. Subsequently, 18 were found to have PAH, 3 had left ventricular dysfunction and 12 had no PAH. At the time of diagnosis, patients in the detection cohort had less significant pulmonary vascular impairment compared to patients from the routine practice cohort [51]. Patients in the detection cohort had a lower mean pulmonary artery pressure and pulmonary vascular resistance index, a higher cardiac output, and were less likely to receive diuretics and warfarin. This screening program provided a strong rationale for timely PAH screening in SSc patients. 

While the diagnostic accuracy of echocardiography alone for the detection of PAH remains suboptimal [13], several algorithms combining clinical features, transthoracic echocardiography, pulmonary function tests, 6 min walking distance (6MWD) test and cardiac biomarkers, namely N-terminal pro-brain natriuretic peptide (NT-proBNP) have been proposed for the detection of SSc patients who should undergo RHC [13,52]. Renal and liver function should also be evaluated at baseline assessment. The combination of different diagnostic modalities such as echocardiography, NT-proBNP and pulmonary function tests provides greater diagnostic accuracy than the use of a single means, preventing unnecessary RHC and correctly identifying patients with mPAP 21–24 mmHg [53]. The DETECT algorithm was validated as highly sensitive (96%) in identifying SSc patients with a higher likelihood of developing PAH and patients with mild or early disease [13]. However, this algorithm has the following major limitations; it is validated in SSc patients with disease duration > 3 years and diffusion capacity of the lung for carbon monoxide (DLCO) <60%, while a high number of patients are referred for RHC (40% to 60%) compared to those that are eventually diagnosed with PAH. SSc patients from the European Scleroderma Trials and Research database with available tricuspid annular plane systolic excursion, systolic PAP and mPAP data were assessed in order to identify complementary parameters for RHC referral [54]. The outcomes of the analysis identified a DLCO < 80% of the predicted value as a highly sensitive marker for the detection of patients with mPAP between 21 and 24 mmHg. 

Composite algorithms such as the Australian Scleroderma Interest Group assessed DLCO, forced vital capacity, NT-proBNP values and PH echocardiographic criteria proposed by the European Society of Cardiology (ESC)/European Respiratory Society (ERS) 2015 PH guidelines [53,55]. Current screening modalities for patients with PAH, however, leave a lot to be desired, as they lack universal validity and usually cannot be applied to all SSc patient groups. An ideal screening method should be cost-effective, highly sensitive, and should significantly limit the number of patients that are unnecessarily referred for RHC. 

According to the 2022 guidelines, cardiopulmonary exercise testing (CPET) should be considered in symptomatic patients with an intermediate echocardiographic probability of PH. More specifically, CPET and maximum oxygen-uptake measurements may be more suitable predictors of PAH prognosis compared to 6MWD [56]. A multicenter study of 173 consecutive SSc patients without diagnosed PAH investigated the value of CPET in detecting SSc-associated PAH [57]. CPET parameters correlated significantly with pulmonary hemodynamics such as pulmonary arterial pressure, transpulmonary pressure gradient and pulmonary vascular resistance. More specifically, oxygen consumption at maximum exercise (peakVO_2_) had the highest sensitivity (87.5%) specificity (74.8%) and therefore diagnostic accuracy for SSc-PAH. Similarly, a study by Santaniello et al. highlighted the capability of CPET to detect the presence of pre-capillary PH in DETECT-positive SSc patients, potentially minimizing the need for RHC [58,59,60].

Serum biomarkers, as non-invasive indicators of endothelial dysfunction, vascular remodeling, inflammation, extracellular matrix deposition and metabolite alterations are promising future tools [61]. The use of omics and non-supervised machine learning methods could help identify clinically significant biomarkers and guide more personalized decision-making, limiting the risk of underdiagnosing PAH, as well as of performing unnecessary RHC [62]. Bauer et al. acquired serum samples from SSc patients with (n = 77) and without PAH (n = 80), randomly selected from the DETECT study. Proteomic screening of 313 proteins was performed, and random forest analysis identified a novel panel of eight proteins (collagen IV, endostatin, insulin-like growth factor binding protein 2, insulin-like growth factor binding protein 7, matrix metallopeptidase-2, neuropilin-1, NT-proBNP and receptor for advanced glycation end products) which discriminated PAH from non-PH SSc patients from the DETECT Cohort [63]. These results were validated in the Sheffield cohort (81.1% accuracy, 77.3% sensitivity, 86.5% specificity) [62]. Nonetheless, association of these proteins with PAH clinical variables was overall weak, and the greatest correlation was noted between NT-proBNP and PVR. Furthermore, Zheng et al. performed a genetic analysis from peripheral blood mononuclear cells of 105 SSc-PAH patients in order to screen potential biomarkers and therapeutic targets for SSc-PAH [64]. Four key genes, including IFIT2, IFIT3, RSAD2 and PARP14 were identified as potentially significant therapeutic targets, as well as indicators for SSc-PAH diagnosis and initiation of specific therapy. These promising results indicate that the integration of omics in the daily clinical practice could play a key role in the development of precision medicine in SSc-PAH. MiRNAs with a well-established role in PAH pathogenesis may also serve as diagnostic and prognostic biomarkers. More specifically, four miRNAs, miR-29, miR-124, miR-140, and miR-204, have demonstrated a conserved pattern of expression in different experimental models and PAH human tissues [65]. Lastly, SSc patients, particularly those with PH, frequently present with concomitant iron deficiency and hypochromic red cells (HRC). A study of 171 SSc patients analyzed based on their iron metabolism indicated that HRC > 2% was a poor prognostic factor of mortality, independently of the presence of PH, especially in combination with DLCO [66].

### 3.2. Screening and Diagnosis of Group 3 PH in SSc

Patients with SSc and group 3 PH constitute a significantly high-risk phenotype associated with poorer prognosis [16] compared to the SSc-PAH group [43,67]. Analyses including clusters of patients with precapillary SSc-PH and different extents of lung fibrosis and hemodynamic impairment showed very poor three-year prognosis in patients with extensive ILD (<50%), severe PH and low diffuse capacity of the lungs (<22%) [68]. There is currently no single non-invasive test that can accurately detect PH in patients with ILD, and thus there is no proposed “standard” approach to assess a patient’s risk for developing PH in ILD. Recently, the modified Delphi study, conducted by a panel of experts in ILD and PH, concluded that an early screening for PH, with a low threshold of suspicion, should be applied [69]. Echocardiography remains the initial diagnostic modality of choice to detect signs of PH among SSc patients with lung involvement and is also useful for the differential diagnosis of left heart disease [70,71,72]. Estimated right ventricular pressure (RVSP) on Doppler echocardiography remains the single most predictive factor of PH, with other indirect echocardiographic markers increasing its diagnostic accuracy. However, RVSP can be difficult to estimate in patients, due to suboptimal views from extensive lung disease. Furthermore, RVSP alone often leads to an overdiagnosis of PH in patients with chronic lung disease [73]. Therefore, RHC remains the gold standard for the diagnosis of group 3 PH [72]. Nonetheless, it is not performed on a routine basis, but, as per the 2022 ERS/ESC PH guidelines [4] it is considered when severe PH is suspected; when the patient’s management will be influenced by RHC results, including consideration of pulmonary vasodilators on an individual case-basis in expert centers, referral for transplantation, exclusion of left heart dysfunction, and inclusion in clinical trials. In addition, pulmonary artery pressure measurement and angiography during RHC, as well as pulmonary artery CTA, can reliably confirm or exclude the presence of chronic pulmonary thromboembolic disease. Chest CT should be considered in the initial evaluation of PH in order to exclude pulmonary embolism and chronic thromboembolic disease [68,69]. Coghlan et al. studied patients who were included in the DETECT study and who had not been diagnosed with PH during initial screening with RHC [74]. Patients were systematically followed and reassessed with RHC during follow-up after 3 years. At follow-up, it was possible to identify PH in almost 25% of patients participating in the study, and PVR was identified as an independent risk factor of disease manifestation.

Pulmonary hemodynamics remain unaffected, due to the pulmonary microvascular reserve, until more than 50% of the microcirculation has been lost due to disease progression [59]. Therefore, screening and detecting exercise PH with exercise RHC in SSc patients may allow for a closer clinical and hemodynamic follow up and earlier detection of PH at rest [75]. Mean PAP values above 30 mmHg during exercise are generally considered early signs of pulmonary vascular remodeling and may help identify patients with early PVD who are at risk of developing PAH [60]. While numerous studies reported an improvement in exercise capacity, resting hemodynamics, and quality of life in SSc patients with exercise PH receiving PAH-targeted therapy, current guidelines do not recommend PAH-specific treatment for exercise PH [75].

Recently, two available non-invasive PH detection scores among patients with ILD have been proposed: one by Bax et al. [76], based on a stepwise echocardiographic approach, and the other by Nathan et al. [77], based on functional parameters. Both scores predicted the presence of PH with sufficient accuracy, and used a validation cohort. However, none of these scores focused on patients with SSc and ILD. Therefore, further studies are needed to compose a diagnostic algorithm targeted to this group of patients.

### 3.3. Discrimination between Group 1 and Group 3 PH in SSc

In general, it can be difficult to differentiate group 1 from group 3 PH. Several criteria pertaining to the extent of lung disease, the hemodynamic profile and the circulatory reserve may help differentiate between the two patient categories [78]. Group 1 patients appear to have a normal or mildly impaired lung function, with FEV1 > 60%, FVC > 70% and low DLCO in relation to obstructive or restrictive changes. On the other hand, group 3 patients have a moderate or severe lung function impairment, with FEV1 < 60%, FVC < 70% and diffusion capacity that corresponds to obstructive or restrictive changes. Respectively, while group 1 patients have moderate-to-severe PH, most of group 3 patients have mild-to-moderate PH, with only a minority presenting with severe PH with PVR > 5 Wood Units [78]. As expected, DLCO is severely reduced and significantly worse in group 3, compared to PAH [79]. Lastly, group 1 patients appear to have indications of a diminished circulatory reserve with reduced oxygen pulse, low minute ventilation (VE) and carbon dioxide production (VCO2) slope (VE/VCO2), mixed venous oxygen saturation at a lower limit and no change or decrease in partial carbon dioxide pressure (PaCO_2_) during exercise. Contrarily, group 3 patients have normal oxygen pulse, normal CO/Vo_2_ slope, mixed venous oxygen saturation above the lower limit and increased PaCO_2_ at exercise.

High-resolution computing tomography (HRCT) may further assist to distinguish the two patient groups [78]. Nonspecific interstitial pneumonia observed in SSc patients is characterized by varying degrees of inflammation and fibrosis. Fibrosis is the predominant finding, without the fibroblastic foci and honeycombing that is observed in usual interstitial pneumonia [80,81]. The abnormalities observed in these patients include irregular, reticular, ground-glass opacities, as well as bronchiectasis and bronchiolectasis. These lesions usually occur in the subpleural regions of the lungs, a detail that helps differentiate nonspecific interstitial pneumonia from usual interstitial pneumonia [80]. These findings were significantly more frequent in SSc patients with precapillary PH than in SSc patients without PAH or ILD. Peripheral interlobular septal thickening and cystic changes, predominantly in the lower lobes, are also findings compatible with SSc-ILD and associated PH [82]. A study of 26 SSc patients by Günther et al. reported centrilobular ground-glass opacities, septal lines and lymph node enlargement as the radiographic triad signs indicative of pulmonary veno-occlusive disease [83]. In addition, an increase in the extent of fibrotic abnormalities found on HRCT is indicative of disease progression [84]. A study of 215 patients from a UK center concluded that extensive ILD, defined as >30% extent of fibrosis on HRCT, or an extent of fibrosis on HRCT of 10–30% with FVC < 70%, was greatly predictive of increased mortality [85]. Therefore, an increase in the extent of fibrotic findings on HRCT may be of great prognostic importance [86,87]. However, while automated post-processing software is able to quantify the extent of fibrotic changes on CT, it is not routinely used in a clinical setting [84]. Furthermore, the enlargement of the main pulmonary artery is a sensitive and specific finding in PH [88]. Dilation of the pulmonary artery (PA) diameter of more than 29 mm or an increased ratio of PA diameter to aortic diameter greater than 1 may be indicative of PH. Moreover, an increased ratio of segmental artery to bronchus diameter > 1 in three or four lobes is highly specific for PH [89]. A dilation of the left or right PA > 18 mm is also indicative of PH, and a predictor of mortality [90]. Mural calcification, increased vascular remodeling, tortuosity and pruning of the peripheral branches are also described in chronic PH [90]. Pericardial effusion with a thickness > 10 mm, especially in the anterior recess, is a strong indicator of PAH and of a poorer prognosis [91].

## 4. Pharmaceutical Management and Effect on Survival

### 4.1. PAH-Targeted Pharmacotherapies in Group 1 PH Associated with SSc

Prompt initiation of PAH-targeted treatment has been demonstrated to greatly improve survival and prognosis [92]. The four main classes of medications prescribed in precapillary PH are ERAs, PDE5 inhibitors, prostacyclin analogues, guanylate cyclase stimulators (GCSs) and Sotatercept, a first-in-class recombinant activin receptor fusion protein [93]. ERAs include non-selective endothelin-A and B receptor antagonists (bosentan and macitentan) and the endothelin-A specific receptor antagonist, ambrisentan. In a double-blind, placebo-controlled study, 32 patients with PAH (primary or associated with SSc) were randomly assigned to receive bosentan (62.5 mg taken twice daily for 4 weeks then 125 mg twice daily) or placebo for a period of 12 weeks [94]. Results indicated that SSc-PAH patients receiving early bosentan therapy presented a stabilization of 6 min walking distance (6MWD). Other nonselective cohort studies assessing bosentan monotherapy in SSc-PAH patients reported an improvement in functional class, 6MWD and hemodynamics following 3–6 months of therapy [95,96,97]. These measures stabilized after 9–12 months of treatment, likely due to the progressive nature of the disease.

PDE5 inhibitors include oral sildenafil, tadafil and vardenafil. A post hoc, subgroup analysis of the double-blind, placebo-controlled SUPER-1 trial demonstrated improvement in exercise capacity, hemodynamics and functional class following 12 weeks of sildenafil therapy in 38 SSc-PAH patients [98]. A retrospective study of SSc-PAH and idiopathic PAH patients analyzed the effects of combination ERA and PDE5 inhibitor therapy [99]. More specifically, sildenafil was added to bosentan monotherapy in patients with clinical deterioration. While functional class and 6MWD showed improvement in idiopathic PAH, no such change was reported in SSc-PAH patients. Furthermore, sildenafil and bosentan interact with one another, leading to an increase in bosentan levels and a reduction in sildenafil concentration. Therefore, SSc-PAH patients reported higher rates of liver toxicity and mortality. The EDITA trial, which included SSc patients with mild PAH commencing ambrisentan, reported a significant reduction in PVR and cardiac index, but not of mPAP, which was the primary endpoint, following 6 months of treatment [100]. These results were further corroborated by a follow-up study assessing the long-term effects of continued ambrisentan therapy compared to no vasodilative treatment [101]. Furthermore, an open-label clinical trial of 24 treatment-naive SSc-PAH patients receiving ambrisentan 10 mg and tadalafil 40 mg daily for 36 weeks exhibited a significant improvement in hemodynamics (55% reduction in PVR and 14% reduction in RV mass), functional class, Borg dyspnea score and quality of life [94]. A subgroup analysis of the AMBITION trial investigating the effect of early ambrisentan–tadafil combination therapy compared to monotherapy with either agent, revealed a lower risk of clinical failure (21% vs. 40%) and a greater improvement in NT-proBNP values and 6MWD with combination treatment [95].

Riociguat, an oral guanylate cyclase stimulator, was assessed in the PATENT-1 and PATENT-2 trials, which included 40 SSc-PAH patients [102]. These patients showed slight improvement in 6MWD, functional class and hemodynamic parameters compared to placebo, following 12 weeks of treatment. Finally, prostacyclin agonists such as parenteral epoprostenol, oral selexipag, parenteral and inhaled treprostinil and iloprost are reserved for high-risk patients [55]. Sotatercept, a novel fusion protein composed of the extracellular domain of the human activin receptor type IIA fused to the Fc domain of the human IgG1, restores balance between the growth-promoting activin growth differentiation factor pathway and the growth-inhibiting BMP pathway [103]. The latter plays a significant role in ensuring endothelial integrity in pulmonary arteries, and mutation in associated genes reduce signaling in this pathway, promoting endothelial impairment and vascular remodeling [104,105]. Sotatercept has been recently approved by the FDA for use in symptomatic PAH patients already on background PAH-targeted therapies [27]. Phase 3 STELLAR trial showed a significant increase in 6MWD at week 24 in the sotatercept arm versus placebo, and an improvement in eight secondary endpoints [106]. Epistaxis, dizziness, thrombocytopenia, telangiectasia, increased hemoglobin and increased blood pressure were the most common adverse effects in the sotatercept arm versus placebo [106].

**Figure 1:**
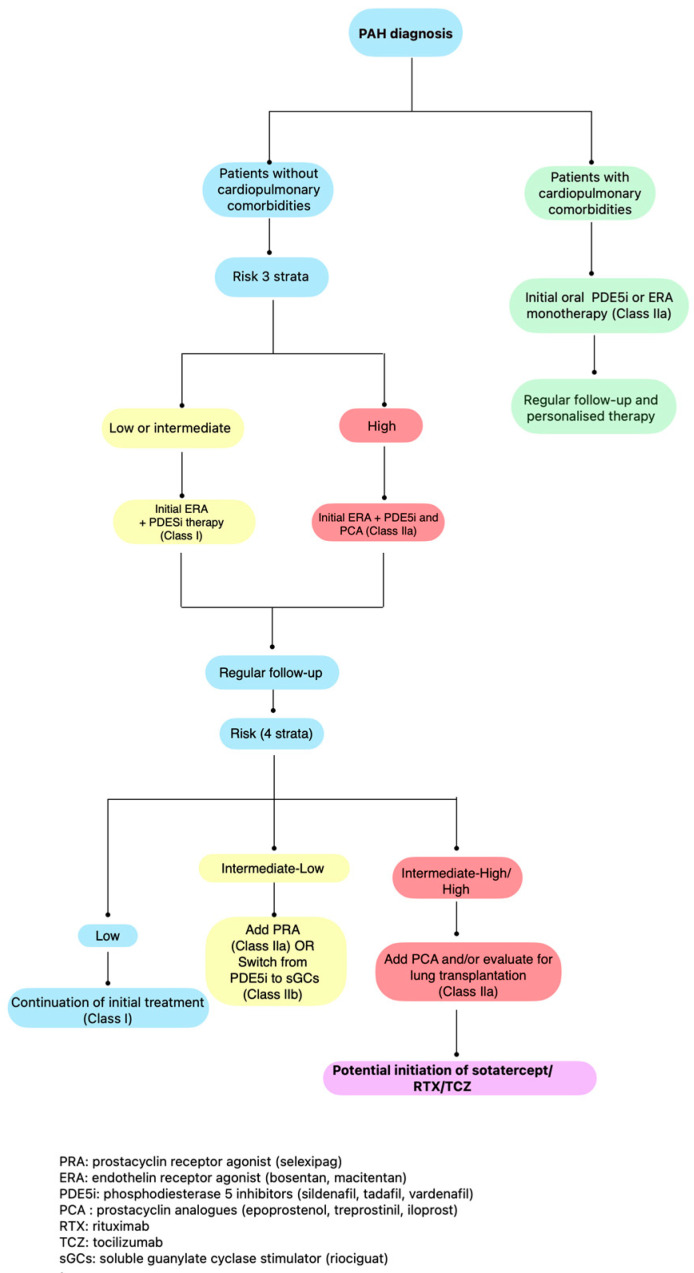
Therapeutic algorithm for managing patients with SSc and PAH according to their risk stratification. Modification of the proposed treatment algorithm according to the 2022 ESC/ERS *Guidelines on Pulmonary Hypertension*.

According to the latest 2022 ESC/ERS PH guidelines, risk stratification at PAH diagnosis and during follow up assessment is fundamental in guiding treatment strategy on the use of PAH-targeted therapies [4]. Patients at low and intermediate risk at baseline should be started on double combination therapy (with a PDE5 inhibitor and an ERA), while for high-risk patients, triple combination therapy with prostanoids is recommended. At follow-up assessment, patients at intermediate–high risk should be escalated to triple therapy, while those at intermediate–low risk should either change the PDE5 inhibitor to riociguat (REPLACE trial) [107] or should be started on selexipag, on top of ERA and PDE5 inhibitor (GRIPHON trial) [108]. The aim of the overall therapeutic management is to keep SSc-PAH patients to as low risk for 1-year mortality as possible. Sotatercept may be added for patients who remain at intermediate–high or high risk at follow-up (Figure 1).

### 4.2. PAH-Targeted Pharmacotherapies in Group 3 PH Associated with SSc

Increasing evidence suggests that timely use of pulmonary vasodilator therapy may also help improve outcomes in group 3 patients with ILD and PH [109]. A meta-analysis by Farmakis et al. demonstrated that PAH-targeted therapies (monotherapy with an ERA or PDE5 inhibitor) improved significantly mPAP, PVR and 6MWD in patients with group 3 PH [110]. Waxman et al. demonstrated the effects of inhaled treprostinil in 325 patients with PH-ILD who were randomly assigned either the prostacyclin agonist or placebo. Overall, treprostinil reduced NT-proBNP levels and improved exercise capacity [111]. Treprostinil palmitil inhalation powder (TPIP) is a dry powder formulation of treprostinil palmitil (TP), which is a pro-drug of treprostinil [112]. It has been designed to provide a sustained release of treprostinil in the lung, potentially enabling a once-daily treatment regimen and higher tolerated doses for patients with PH-ILD. A phase I study investigating the safety of the single-dose regimen indicated that 70.8% of all participants (24 overall) receiving TPIP experienced a treatment-emergent adverse event, with the most common being cough, dizziness and throat irritation [112]. Similarly, mild adverse events were reported in most patients receiving a multi-dosage regimen, whereas moderate adverse events were noted in seven patients. TPIP is an investigational product and has not been approved for use in clinical practice.

An open-label study of 111 SSc-PH patients receiving epoprostenol therapy for 12 weeks revealed an improvement in exercise capacity and hemodynamics compared to conventional therapy [113]. An open-label extension of the same study failed to provide long-term outcomes of these patients, due to technical limitations, but reported a 3-year survival rate of 52%, which was higher than previous cohorts [114]. Early initiation of aggressive therapy with prostanoids in group 3 patients correlated with better survival rates [114].

### 4.3. Impact of PAH-Targeted Therapies on Survival

Precapillary SSc-PH is estimated to affect 15–18% of patients, with SSc-PAH being the prevalent form, with an incidence of 6–9% [115]. A study by Hassan et al. concluded that transplant-free survival has significantly improved for patients with SSc-PAH over the last decade (estimated 5-year rate: 60% vs. 37% in the previous decade) [116]. This improvement was mainly attributed to earlier diagnosis, better baseline clinical and hemodynamic characteristics, and use of disease-specific upfront combination therapy, but should be interpreted with caution, as lead-time bias could affect survival [49]. Age > 60 years, male sex, systolic blood pressure ≤ 110 mmHg, pericardial effusion, PVR > 32 Woods, DLCO > 39%, poor functional status, 6MWD < 165 m and BNP > 180 pg/mL have been identified as significant predictors of mortality in SSc-PAH patients [14,117,118,119].

Over the past decades, the availability of standardized risk stratification methods, as well as the prompt initiation of combination therapy, have generally improved the prognosis of SSc-PAH patients (Table 1) [120]. Upfront combination therapy is strongly supported by the results of the AMBITION and GRIPHON studies in PAH patients [4,108]. A study of 504 SSc-PH patients enrolled in the Johns Hopkins Pulmonary Hypertension Center Registry by Hassan et al. compared survival rates of patients diagnosed between 1999 and 2010 and those diagnosed between 2010 and 2021 [116]. In total, 308 (61%) patients were diagnosed with group 1 PH, 43 (9%) with group 2 PH, and 151 (30%) with group 3 PH. Interestingly, group 1 patients diagnosed between 2010 and 2021 had significantly better clinical and hemodynamic characteristics at baseline, were more likely to have initiated PAH-targeted combination therapy at an early disease stage, and had a nearly 4-year increase in median transplant-free survival rate compared to patients diagnosed between 1999 and 2010. Patients with group 2 and 3 PH did not differ in terms of baseline clinical, hemodynamic or survival characteristics between the two-time intervals, and continued to have dismal outcomes.

Another study by Hachulla et al. compared survival rates among 167 SSc patients diagnosed with PAH from 2006 to 2011 with those of 139 patients diagnosed from 2012 to 2017 [123]. Notably, more patients aged ≤ 70 years received combination ERA/PDE5i therapy within the first 4 months in 2012–2017 compared to those diagnosed and treated from 2006 to 2011 (42.9% vs. 19.5%). The gradual improvement in survival was attributed to the earlier diagnosis of PAH, as well as to the prompt initiation of aggressive treatment, particularly epoprostenol/sildenafil and ambrisentan/tadalafil combination therapy. Similarly, improved survival rates were also reported by Khanna et al. in CTD-PAH patients treated after 2010, compared to those treated before 2010 [12]. While overall risk of death for CTD-PAH patients remains higher than that of PAH attributed to other causes, increased screening and novel treatment strategies were identified as the main factors affecting morbidity and mortality. Moreover, results from the COMPERA registry indicated an increase in the use of early combination therapy from 10% in PAH patients diagnosed in 2010 to 25% in patients diagnosed in 2019. Similarly, the percentage of patients receiving combination therapy 1 year after diagnosis increased from 27.7% to 46.3%. However, comparing the 2010–2014 and 2015–2019 periods, 1-year survival estimates were found to be similar, and there was a slight but non-significant improvement in 3-year survival rates [17].

In addition, a study of 364 SSc-PAH and 1589 non-SSc-PAH patients indicated that aggressive SSc-PAH treatment from disease onset improved outcomes, regardless of the presence of concomitant ILD [109]. More specifically, upfront combination therapy was used in 59.8% and 61.7% of patients with and without ILD, respectively. The five-year transplant-free survival rate was 41.1% in SSc-PAH patients and 93.9% in non-PAH patients, whereas the global survival of SSc-PAH remained unaffected by the severity of ILD. On the other hand, mortality was greatly affected by clinical, pulmonary function tests and hemodynamic factors, as well as by up-front combination PAH-targeted therapy. The poorer pulmonary capacity and hemodynamics observed in SSc-PAH patients with concomitant ILD did not translate into worse transplant-free survival [9,127].

In contrast to SSc-PAH patients, no significant improvement was reported for group 3 SSc-PH patients, with mortality rates remaining high (3-year survival rate 35%) [67]. A study of 128 SSc patients (66 SSc-PH-ILD and 62 SSc-PAH) aimed to compare survival rates and response to treatment between the two patient groups [128]. At baseline, SSc-PH-ILD patients had a less severe hemodynamic impairment and DLCO compared to the SSc-PAH group. Despite the fact that both groups had similar hemodynamic improvement, SSc-ILD-PH patients exhibited improvement in WHO functional class less frequently compared to SSc-PAH patients, and had increased mortality at 1, 2 and 3 years.

### 4.4. Immunosuppressive Therapy

The potential role of immunosuppression using targeted DMARDs in the therapeutic management of precapillary PH in SSc patients is currently being investigated. A study of 755 SSc patients from the EUSTAR project with PAH reported that targeted therapies, specifically rituximab and tocilizumab, decreased significantly the risk of PAH worsening and mortality [126].

Due to the possible role of B cells in the pathogenesis of SSc and SSc-ILD [129], rituximab is being studied for its effects on SSc-ILD. A study of 30 SSc patients with elevated pulmonary artery systolic pressure (PASP) receiving rituximab for ILD indicated that B-cell depletion therapy correlated with a significant decrease in PASP, as well as an increase in forced vital capacity and stabilization of DLCO. Concomitantly, the improvement in lung function was accompanied by a considerable increase in 6MWD and in the left ventricular ejection fraction [125]. Consequently, rituximab appears promising for the management of SSc- ILD.

Furthermore, a multicenter, double-blinded, randomized trial of 57 SSc-PAH patients demonstrated that rituximab was potentially effective in improving 6MWD and hemodynamic parameters, with acceptable tolerance and safety profile [124]. In general, the role of DMARDs in the therapeutic management of SSc-PAH remains greatly overlooked, and further studies are needed to validate their efficacy and safety.

Finally, the existing literature indicates that IL-6 is elevated in the serum of PH patients and higher concentrations correlate with a worse lung function [130]. Therefore, anti-IL-6 therapies are being investigated for their safety and efficacy in early SSc-ILD [131]. Tocilizumab, and anti-IL-6 antibody had no statistically significant effect on skin sclerosis, but preserved lung function over 48 weeks in early SSc-ILD patients, participating in a phase 3 RCT [132,133]. Further studies are needed to corroborate these results and to investigate drug safety in SSc patients.

In light of these promising results, the notion that targeting B cells responsible for producing pathogenic SSc autoantibodies could inhibit chronic inflammation and consequent fibrosis is gathering increasing interest [134]. Moreover, changes in Treg population and, more specifically, an overactive Th17 response, are also considered responsible mechanisms of fibrosis and tissue damage in SSc patients [135]. It is possible that specific CAR-Tregs targeting skin and lung antigens could potentially induce tolerance in early and late stages of disease progression, reducing inflammation and promoting tissue repair [134]. As pleural disorders (nonmalignant pleural effusion) are over-reported in CAR-T recipient patients, it remains unknown whether SSc-PAH patients could undergo such treatment [136]. While fluid retention in CAR-T patients could partly explain these complications, they may also result from cytokine release syndrome and capillary leak, although evidence concerning volume overload manifestations is scarce [134].

Lastly, the added benefit of increased use of corticosteroid therapy in SSc-PAH is controversial. Corticosteroid medication may be beneficial in CTD-PH, even in cases of echocardiographically assessed PH or low general CTD activity. Real-world data indicate that corticosteroid therapy (oral or pulse therapy) is beneficial for CTD-PH patients, either on its own or in combination with conventional vasodilators, as it improves hemodynamic evaluations and pulmonary function [137,138]. RCTs are lacking, though, to confirm these observations, and therefore a cautious use is recommended.

### 4.5. Antifibrotic Treatment

Nintedanib, a small-molecule tyrosine kinase inhibitor (TKI), has been investigated for its effects in SSc patients with ILD. Patients from the SENSCIS trial were randomized to receive 150 mg of oral nintedanib twice daily, with or without mycophenolate, or placebo, for at least 52 weeks. Νintedanib improved lung function and hindered the progression of ILD in patients with SSc-ILD, regardless of the mycophenolate therapy [139,140]. Despite this evidence that combination therapy with immunosuppressive (tocilizumab, rituximab) and antifibrotic (nintedanib) agents could provide a new effective treatment plan for SSc-ILD, no clear recommendations regarding the initiation and timing of such therapy have been announced [141].

Pirfenidone, an antifibrotic and anti-inflammatory drug that regulates TNF and TGF-β pathways, inhibiting fibroblast proliferation and collagen deposition, has also revolutionized pulmonary fibrosis management [142]. Pirfenidone treatment has been associated with a delay in the decline of forced vital capacity (FVC) and disease progression in patients with pulmonary fibrosis [143,144]. Nonetheless, drug use has been limited to idiopathic pulmonary fibrosis, and pirfenidone has not been approved for other forms of ILD [145]. The RELIEF study of 127 ILD patients with a progressive functional decline demonstrated that pirfenidone-treated patients had a significantly lower deterioration in FVC%, compared to placebo [146]. However, as the trial was ended prematurely due to slow recruitment, no definite conclusions can be drawn regarding FVC changes. A double-blind, randomized, placebo-controlled study of 34 SSc-ILD patients reported stabilization or improvement in FVC in 94.1% of subjects receiving 6 months of pirfenidone and in 76.5% of subjects receiving placebo [147]. Major limitations of the study are the small sample size and the short follow-up period.

Overall, the enhancement of the therapeutic armamentarium with antifibrotic drugs which ameliorate the physical course of progressive ILD in patients with SSc may also have an important effect on the improvement of outcomes in patients with SSc-ILD, regarding both the incidence and mortality of PH-ILD. However, such expectations remain to be confirmed in large, longitudinal population-based studies over the coming years.

### 4.6. Atrial Septostomy

For individuals with PAH unresponsive to medical therapy, interventional and surgical alternatives remain effective. A common method is Atrial Septostomy (AS). With increased pressure in the right atrium, AS permits right-to-left shunting and, therefore, offloading of the right atrium at the expense of hypoxia. Various transcatheter techniques are proposed for AS. The recommended method is stepwise balloon dilatation [148]. A Brockenbrough needle is used to puncture the interatrial septum, and an up-to-18 mm Hg balloon catheter is then inserted, and gradually inflated, until there is a 10% fall in arterial oxygen saturation or an increase in left ventricular end-diastolic pressure. Another technique is called blade balloon AS, in which the oval fossa’s limbs are sliced as it is steered away from the anterior aortic root, using a 5 to 15 mm blade [149]. AS has been associated with a better exercise tolerance, cardiac index and New York Heart Association (NYHA) functional class in individuals who are not responding to medication [150]. Patient selection should be carefully made. AS is not beneficial for patients with oxygen saturation below 90%, severe RV failure on cardiorespiratory support, low cardiac output, or high right atrial pressure (mean > 20 mmHg) [151].

## 5. Conclusions

Precapillary PH is a severe complication among patients with SSc that skyrockets mortality rates. Although, prognosis differs among the distinct PH groups, death rates remain significantly high, with additional cardiovascular burden related to SSc further impacting survival. The implementation of screening programs using non-invasive markers for the early detection of patients at risk of PH and the timely referral to PH expert centers for RHC and final diagnosis and discrimination of PH subgroup, and risk stratification at the time of PAH diagnosis, coupled with the prompt initiation of aggressive/up-front combination therapy, as well as the emerging role of immunosuppressive/antifibrotic regimens in patients with ILD, could justify a cautious optimism for the management of this devastating disease.

## Figures and Tables

**Table 1:** Studies reporting improved survival rates.

Intervention	Study Type	Study Population	Outcomes	Reference
Epoprostenol therapy for 12 weeks	Uncontrolled open-label 3-year extension study following an initial 12-week RCT	111 SSc-PAH patients	- 52% 3-year survival rate - improved exercise capacity and hemodynamic parameters with therapy	Badesch et al., 2009 [114]
Prostanoid therapy for 6 months	Observational study (retrospective cohort study)	99 SSc-PAH patients	- 50% and 60% survival rates - survival rates improved moderately with aggressive prostanoid therapy	Volkmann et al., 2014 [121]
Ambrisentan 10 mg and tadalafil 40 mg combination therapy or monotherapy with either agent until first clinical failure event.	Post hoc analysis of an event-driven, double-blinded RCT	118 SSc-PAH patients and 180 CTD-PAH patients	- initial combination therapy resulted in a reduction in the risk of clinical failure versus pooled monotherapy of 56% in SSc-PAH patients. - 15% worsening of 6MWD (determinant of poor prognosis) observed in (31%) on combination therapy compared to 44% on monotherapy in the SSc-PAH group	Coghlan et al., 2017 [122]
Epoprostenol/sildenafil and ambrisentan/tadalafil combination therapy within 4 months of diagnosis.	Observational study	306 SSc-PAH patients	- more patients aged ≤ 70 years received combination therapy within 4 months in 2012–2017 compared to 2006–2011 (42.9% vs. 19.5%)	Hachulla et al., 2020 [123]
Rituximab for 24 weeks.	Multicenter, double-blinded RCT	57 SSc-PAH patients	- improvement in hemodynamic parameters	Zamanian et al., 2021 [124]
Baseline pulmonary vasodilator combination therapy for 5 years.	Observational study	364 SSc-PAH and 1589 non- SSc-PAH patients	- 5-year transplant-free survival rate 41.1% in SSc-PAH patients and 93.9% in non- SSc-PAH patients - death increased by clinical, PFT, and hemodynamic factors and decreased with combination therapy	Guillén-Del-Castillo et al., 2022 [109]
Rituximab for 25.3 ± 2.4 months	Observational study	30 SSc patients with increased PASP	- significant decrease in PASP -improved lung function - improved hemodynamic parameters	Garzanova et al., 2022 [125]
Rituximab, tocilizumab for at least 30 days.	Observational study	755 SSc patients with precapPH	- reduced risk of mortality and precapPH worsening	Bruni et al., 2023 [126]
